# The Multidimensional Assessment of Interoceptive Awareness, version 2 (MAIA-2): psychometric properties in a Dutch non-clinical sample

**DOI:** 10.1186/s40359-024-01553-8

**Published:** 2024-01-29

**Authors:** Mia Scheffers, Jaisey Coenen, Janet Moeijes, Albertine de Haan, Jooske van Busschbach, Tina Bellemans

**Affiliations:** Department of Human Movement and Education, Windesheim University of Applied Sciences, Zwolle, the Netherlands

**Keywords:** Interoceptive awareness, Psychometric properties, Dutch version, Exploratory factor analysis, Confirmatory factor analysis

## Abstract

**Background:**

Interoceptive awareness is a multidimensional construct that refers to the sensation, interpretation, and integration of signals within the body. There is increasing evidence that problems with interoceptive awareness form an important component of mental health problems. The Multidimensional Assessment of Interoceptive Awareness 2 (MAIA-2) is presently the most used self-report questionnaire to measure interoceptive awareness. The aim of the present study is to psychometrically evaluate the Dutch version of the MAIA-2.

**Method:**

The psychometric properties of the MAIA-2-NL were examined in a non-clinical sample of 1054 participants aged between 18 and 83. Internal consistency and test-retest reliability were investigated. Factor structure was examined by exploratory factor analysis (EFA), followed by confirmatory factor analysis (CFA).

**Results:**

Internal consistency was good, with McDonald’s omega (ω) ranging from 0.67 to 0.89. Test-retest reliability was moderate to good, with intraclass correlation coefficients (ICC) ranging from 0.67 to 0.79. Factor analyses suggested a six-factor structure, combining the original subscales Noticing with Emotional awareness and Self-regulation with Body listening. However, a CFA based on the original eight factors showed a somewhat better fit than the CFA based on six factors.

**Conclusion:**

The MAIA-2-NL is a reliable and valid instrument to measure interoceptive awareness in healthy Dutch adults. We recommend to maintain the original 37 items.

**Supplementary Information:**

The online version contains supplementary material available at 10.1186/s40359-024-01553-8.

## Introduction

Interoception is the process by which the nervous system senses, interprets, and integrates signals originating from within the body [1].These signals include physical sensations related to internal organ functions such as heartbeat, respiration, satiety and the autonomic nervous system activity related to emotions [2]. Many of these remain unconscious, but some are conscious or are potentially accessible to consciousness. Interoceptive awareness refers to this conscious level of interoception [3].

Interoceptive awareness helps the organism to maintain homeostasis and is thus crucial for survival [4]. Moreover, it is thought to be connected to self-regulation and emotion-regulation [2, 5]. It is suggested that interoceptive awareness is often problematic in patients with mental disorders, such as mood disorders [5], posttraumatic stress disorder [6], somatic symptom disorders [7], and eating disorders [8, 9]. While most studies have been conducted in patients with specific diagnoses, problems in interoceptive awareness might be regarded as a transdiagnostic factor, a common vulnerability across multiple disorders [10]. Awareness of physical sensations may be upregulated or downregulated in patients with mental disorders [11].

Interoceptive awareness has been measured by different self-report questionnaires, such as the Porges Body Perception Questionnaire (BPQ) [12], the Scale of Body Connection (SBC) [13, 14], the Somatic Awareness Questionnaire (SAQ) [15, 16], and the Multidimensional Assessment of Interoceptive Awareness (MAIA), version 1 and 2 [3, 17]. Interoceptive awareness is closely related to interoceptive sensitivity, described by Forkman et al. [18] as a dispositional tendency to be internally focused. This term thus captures beliefs about body sensations usually assessed with self-report questionnaires. However, the term interoceptive sensibility does not differentiate clearly an anxiety and hypervigilance-driven attention style versus a more mindful, adaptive and potentially healthy attention style. The MAIA-2 has been designed to differentiate between anxiety-driven and mindful attention styles towards interoceptive cues, a distinction of key clinical importance [3, 19]. Presently, the MAIA-2 is the most used self-report questionnaire, covering different components of interoceptive awareness. The MAIA-2 has been translated and psychometrically evaluated in several languages, including French [20], Turkish [21], Chinese [22], Norwegian [23], Ukrainian [24], and German [5]. These studies used non-clinical samples except for the study conducted by Eggart et al. [5], who provided psychometric values for the MAIA-2 in a group of people with Major Depressive Disorder. The above-mentioned studies, as well as studies using the first version of the MAIA, revealed varying results regarding factor structure and internal consistency of the subscales.

To date, the MAIA-2 has not been translated and psychometrically evaluated in Dutch. The SAQ [15, 16], as well as the SBC [13, 14], have been used in studies in the Netherlands [25, 26], but both lack thorough psychometric evaluation. Furthermore, these questionnaires do not cover the multidimensional concept of interoceptive awareness as applied in the MAIA-2, which we deem preferable for an instrument to be used in a broad array of mental disorders. Therefore, we refrained from further evaluation of the SAQ and SBC and selected the MAIA-2 for psychometric evaluation in a convenience sample of Dutch adults.

The central aim of this article is to evaluate the psychometric properties of the Dutch version of the MAIA-2 (MAIA-2-NL) in a non-clinical sample. This step may be regarded as the first necessary step, after which psychometric evaluation in different target groups should follow.

## Method

### Participants

Data were collected in a non-clinical sample of Dutch adults (*n* = 1054) aged between 18 and 83. The sample consisted of 331 (31.4%) men and 723 (68.6%) women. Descriptive statistics are shown in Table 1.

**Table 1:** Demographic characteristics of the sample

		Frequency (%)	Mean (SD)	Range (min-max)
Total (n)		1054 (100)		
	Men	331 (31.4)		
	Women	723 (68.6)		
Education level^*^				
	Higher education	652 (61.9)		
	Medium education	370 (35.1)		
	Lower education	32 (3.0)		
Age			35.15 (15.74)	18–83
	Men (331)		37.51 (16.45)	18–83
	Women (723)		34.07 (15.30)	18–82
BMI (*n* = 1033)			24.34 (4.01)	16.00-44.69
	Men (327)		24.95 (3.81)	16.11–42.28
	Women (706)		24.09 (4.10)	16.00-44.69

* Based on the International Standard Classification of Education

### Procedure

Data collection took place in agreement with the Medical Ethical Committee of the University Medical Center Groningen. Students enrolled in a bachelor psychomotor therapy research course in 2019 collected data from their personal network, resulting in a convenience sample of Dutch adults. Participation was voluntary, and data were analysed anonymously. No participatory incentives were offered. All participants were informed of the study’s purpose, method, and voluntary and anonymous nature of participation before they entered the study. Data was collected using computerized questionnaires through a secure online survey platform (Formdesk). To assess temporal reliability 109 respondents (Men = 30, Women = 79, Mean age = 43.78 (SD = 17.86), Mean BMI = 24.79 (SD = 4.31)) completed the questionnaire again after 14 days.

### Measures

The MAIA-2 is a self-report questionnaire developed by Mehling et al. [3] (see Table 2). It is a 37-item scale that consists of eight subscales: Noticing, Not-distracting, Not-worrying, Attention regulation, Emotional awareness, Self-regulation, Body listening and Trusting. Subscale scores are calculated by taking the arithmetic mean of the items on each scale. Items are scored on a 6-point Likert scale, ranging from 0 (never) to 5 (always). Higher scores indicate more interoceptive awareness [3].

A forward-backward translation based on the original English version of the MAIA-2 was made following a recommended five-step procedure [27]: (1) Two forward translations were performed independently by two native Dutch speakers, with proficiency in English and with knowledge of the concept of interoceptive awareness and its meaning in practice; (2) The two versions were then compared item-by-item, and discrepancies were resolved. (3) Two independent professional bilingual translators, naive to the construct of interoceptive awareness, performed a back-translation into English; (4) Discrepancies between the back-translations and the original English version were discussed and resolved; (5) This resulted in a pre-final Dutch version. In the next step, a pilot study was conducted among 41 respondents to examine the usability and comprehensibility. This led to minor adjustments in the wording of some items, resulting in the final version used in the present study (see Table S1).

**Table 2:** Multidimensional Assessment of Interoceptive Awareness, English version*

1.a	When I am tense, I notice where the tension is located in my body
2.a	I notice when I am uncomfortable in my body
3.a	I notice where in my body I am comfortable
4.a	I notice changes in my breathing, such as whether it slows down or speeds up
5.b	I ignore physical tension or discomfort until they become more severe (R)
6.b	I distract myself from sensations of discomfort (R)
7.b	When I feel pain or discomfort, I try to power through it (R)
8.b	I try to ignore pain (R)
9.b	I push feelings of discomfort away by focusing on something (R)
10.b	When I feel unpleasant body sensations, I occupy myself with something else so I don’t have to feel them (R)
11.c	When I feel physical pain, I become upset (R)
12.c	I start to worry that something is wrong if I feel any discomfort (R)
13.c	I can notice an unpleasant body sensation without worrying about it
14.c	I can stay calm and not worry when I have feelings of discomfort or pain
15.c	When I am in discomfort or pain I can’t get it out of my mind (R)
16.d	I can pay attention to my breath without being distracted by things happening around me
17.d	I can maintain awareness of my inner bodily sensations even when there is a lot going on around me
18.d	When I am in conversation with someone, I can pay attention to my posture
19.d	I can return awareness to my body if I am distracted
20.d	I can refocus my attention from thinking to sensing my body
21.d	I can maintain awareness of my whole body even when a part of me is in pain or discomfort
22.d	I am able to consciously focus on my body as a whole
23.e	I notice how my body changes when I am angry
24.e	When something is wrong in my life, I can feel it in my body
25.e	I notice that my body feels different after a peaceful experience
26.e	I notice that my breathing becomes free and easy, when I feel comfortable
27.e	I notice how my body changes when I feel happy / joyful
28.f	When I feel overwhelmed, I can find a calm place inside
29.f	When I bring awareness to my body, I feel a sense of calm
30.f	I can use my breath to reduce tension
31.f	When I am caught up in thoughts, I can calm my mind by focusing on my body/ breathing
32.g	I listen for information from my body about my emotional state
33.g	When I am upset, I take time to explore how my body feels
34.g	I listen to my body to inform me about what to do
35.h	I am at home in my body
36.h	I feel my body is a safe place
37.h	I trust my body sensations

R = reversely scored; a = subscale Noticing; b = subscale Not-distracting; c = subscale Not-worrying; d = subscale Attention regulation, e = subscale Emotional awareness, f = subscale Self-regulation, g = subscale Body listening; h = subscale Trusting

^*^ original version in English; for Dutch version, see Table S1

### Data analysis

SPSS 28 for Windows was used for statistical analyses. Factor structure was examined by exploratory factor analysis (EFA) and confirmatory factor analysis (CFA). The sample was randomly split into two halves, one of which was analysed with EFA and the other with CFA. Reliability was assessed using both McDonald’s omega and Cronbach’s α. McDonald’s omega was used because the assumption of tau-equivalency is not accepted [28]. Cronbach’s α was reported to allow comparisons with other validation studies. In addition, there is no universally accepted guideline for acceptable levels of omega reliability, but they need to meet the same standards as Cronbach’s α [29]. Thus, an omega ≥ 0.70 is regarded as good [30]. Test-retest reliability was established by intraclass correlation (ICC). A two-way mixed model for absolute agreement was used [31]. An ICC between 0.50 and 0.75 was considered moderate, between 0.75 and 0.90 good, and ≥ 0.90 excellent [32].

For the EFA, Maximum Likelihood with oblique rotation was used as factor extraction method. Kaiser’s criterion, requiring factors with eigenvalues of at least 1, was adhered to [33]. The number of factors retained was based on interpreting the scree plot [34] and parallel analysis [35]. Cross-loadings were defined as an item that loads at > 0.32 on two or more factors [36].

The other half of the sample (*n* = 527) was used for confirmatory analysis (CFA) to evaluate the adequacy of the proposed factor structure following EFA [37–39]. Mplus Version 8.0 was used [40]. Each index type provides different information about model fit [37]. Therefore, a broad range of indices was reported, including root mean square error of approximation (RMSEA) [41, 42], standardized root mean square residual (SRMR) [43], Comparative Fit Index (CFI), and Tucker Lewis index (TLI) [37, 40, 44].

## Results

Table 3 shows the descriptive statistics forthe subscales, along with internal consistency, both from the entire sample and separately for men and women. Table 4 shows the descriptive statics and internal consistency for both the Exploratory Factor Analysis (EFA) and Confirmatory Factor Analysis (CF) samples, as well as the test-hertest sample. The internal consistency (ω) of all subscales of the MAIA-2 NL was moderate to good with ω = 0.67 to 0.89. The MAIA-2 subscales showed moderate to good reliability over time, with intraclass correlation coefficients (ICC) varying from 0.67 to 0.79.

**Table 3:** Descriptive Statistics, internal consistency, and sex differences

	Complete sample (*n* = 1054)			Men (*n* = 331)	Women (*n* = 723)	Differences men and women	
	Mean (SD)	α	ω	Mean (SD)	Mean (SD)	t (df = 1052)	
Noticing	3.37 (0.86)	0.67	0.67	3.15 (0.92)	3.46 (0.81)	5.33*	
Not-distracting	2.17 (0.87)	0.80	0.81	2.19 (0.85)	2.16 (0.88)	− 0.53	
Not-worrying	3.35 (0.82)	0.78	0.78	3.45 (0.81)	3.30 (0.83)	− 0.27	
Attention regulation	2.88 (0.85)	0.86	0.86	2.96 (0.81)	2.85 (0.86)	-1.94	
Emotional awareness	3.50 (0.84)	0.80	0.79	3.32 (0.87)	3.59 (0.81)	4.87*	
Self-regulation	2.65 (0.97)	0.81	0.82	2.71 (0.94)	2.62 (0.99)	-1.37	
Body listening	2.38 (1.06)	0.82	0.82	2.32 (1.04)	2.41 (1.07)	1.22	
Trusting	3.63 (1.02)	0.88	0.89	3.80 (0.97)	3.55 (1.04)	-3.69*	

* = *p* <.001

**Table 4:** Descriptive Statistics EFA and CFA samples, and test-retest

	Sample EFA (*n* = 527)			Sample CFA (*n* = 527)			Test-retest sample (*n* = 109)	
	Mean (SD)	α	ω	Mean (SD)	α	ω	Mean (SD)	ICC
Noticing	3.39 (0.84)	0.66	0.67	3.38 (0.90)	0.70	0.71	3.37 (0.89)	0.79
Not-distracting	2.14 (0.89)	0.81	0.81	2.13 (0.90)	0.81	0.82	2.12 (0.79)	0.67
Not-worrying	3.38 (0.80)	0.77	0.78	3.34 (0.85)	0.80	0.81	3.42 (0.69)	0.69
Attention regulation	2.89 (0.82)	0.85	0.85	2.84 (0.87)	0.86	0.86	2.90 (0.89)	0.74
Emotional awareness	3.52 (0.83)	0.79	0.79	3.49 (0.86)	0.81	0.81	3.48 (0.90)	0.73
Self-regulation	2.66 (0.96)	0.80	0.81	2.62 (0.98)	0.82	0.83	2.79 (0.96)	0.73
Body listening	2.38 (1.02)	0.80	0.80	2.37 (1.07)	0.82	0.82	2.61 (1.01)	0.68
Trusting	3.66 (1.02)	0.88	0.89	3.61 (1.06)	0.89	0.89	3.79 (0.93)	0.70

EFA revealed a six-factor structure as best solution, with the items from the original subscales Noticing (items 1 to 4) and Emotional awareness (items 23 to 27) as well as the subscales Self-regulation (items 28 to 31) and Body listening (items 32 to 34) taken together as one factor. The other items are loaded at four factors, which is in accordance with the subscales as proposed by Mehling et al. [3], see Table 5.

**Table 5:** Exploratory Factor Analysis

Item	Factor 1	Factor 2	Factor 3	Factor 4	Factor 5	Factor 6
1.a	− 0.066	− 0.013	− 0.043	0.026	**0.541**	0.041
2.a	− 0.007	0.103	− 0.071	− 0.049	**0.494**	− 0.097
3.a	0.025	0.076	− 0.019	0.076	**0.427**	0.102
4.a	− 0.072	0.135	− 0.008	0.011	**0.354**	− 0.022
5.b	0.042	− 0.024	**0.448**	0.122	0.150	− 0.018
6.b	0.017	0.007	**0.550**	0.019	− 0.085	− 0.015
7.b	− 0.022	− 0.043	**0.617**	− 0.047	− 0.062	0.047
8.b	0.053	0.035	**0.718**	− 0.074	0.005	0.026
9.b	− 0.070	0.088	**0.773**	− 0.034	− 0.039	− 0.072
10.b	− 0.015	0.056	**0.786**	0.039	− 0.062	0.013
11.c	− 0.033	− 0.078	0.136	**0.714**	0.017	0.032
12.c	0.049	− 0.037	0.088	**0.729**	0.037	− 0.068
13.c	0.013	0.088	− 0.119	**0.538**	0.090	0.007
14.c	0.076	0.130	− 0.090	**0.641**	− 0.066	0.042
15.c	0.151	0.052	0.019	**0.497**	− 0.100	0.021
16.d	− 0.044	**0.509**	0.047	0.051	− 0.053	0.174
17.d	0.051	**0.583**	0.028	0.032	0.081	0.024
18.d	0.089	**0.481**	− 0.009	0.161	0.113	0.056
19.d	0.009	**0.784**	0.048	− 0.009	0.023	0.020
20.d	0.019	**0.705**	0.038	0.053	0.080	0.080
21.d	0.130	**0.478**	− 0.009	0.161	0.113	0.056
22.d	0.125	**0.517**	− 0.007	0.060	0.156	0.103
23.e	− 0.013	0.106	− 0.017	0.039	**0.562**	− 0.017
24.e	− 0.002	0.065	0.039	− 0.184	**0.613**	− 0.124
25.e	0.074	− 0.145	0.004	− 0.005	**0.579**	0.248
26.e	0.057	− 0.035	0.030	0.030	**0.663**	0.163
27.e	0.115	− 0.128	− 0.002	0.011	**0.746**	0.077
28.f	0.131	0.027	− 0.039	0.157	− 0.069	**0.571**
29.f	0.075	0.003	− 0.002	0.119	0.103	**0.634**
30.f	− 0.058	0.168	0.014	0.011	0.035	**0.607**
31.f	− 0.075	0.205	− 0.057	0.029	− 0.049	**0.685**
32.g	0.015	0.089	0.118	− 0.244	0.197	**0.471**
33.g	0.030	0.130	0.061	− 0.243	0.154	**0.475**
34.g	0.166	0.147	0.045	− 0.125	0.105	**0.386**
35 h	**0.885**	0.001	− 0.033	0.003	− 0.067	− 0.013
36.h	**0.981**	0.038	0.009	− 0.010	− 0.060	− 0.052
37.h	**0.654**	0.109	0.047	0.043	0.089	0.025

a = subscale Noticing; b = subscale Not-distracting; c = subscale Not-worrying; d = subscale Attention regulation, e = subscale Emotional awareness, f = subscale Self-regulation, g = subscale Body listening; h = subscale Trusting

CFA on the six factors resulting from the EFA (see Table 5) provided an acceptable fit. However, CFA with the original eight factors resulted in a better fit (see Table 6).

**Table 6:** Goodness of fit index values for CFA

	χ²	df	RMSEA (90% CI)	SRMR	CFI	TLI
6 Factors	2324	619	0.072 (0.069 − 0.075)	0.069	0.781	0.765
8 factors	1662	601	0.058 (0.055 − 0.061)	0.057	0.864	0.849

χ2 = chi square; df = degrees of freedom; RMSEA = root mean square error of approximation; 90% CI = 90% confidence interval of the RMSEA; SRMR = standardized root mean square residual; CFI = comparative fit index; TLI = Tucker Lewis index

## Discussion

The central aim of this study was to evaluate the psychometric properties of the Dutch version of the MAIA-2 (MAIA-2-NL) in a large non-clinical sample of Dutch adults. Our analyses show good internal consistency and reliability over time for all eight MAIA-2-NL subscales. EFA and CFA points towards a six-factor structure instead of the eight-factor structure from the original English version [3]. However, the CFA reveals better fit indices for an eight factor solution than for a six factor solution.

A six-factor structure was also established as best solution in the French [20] and Turkish [21] versions of the MAIA-2. However, in the French version, the subscales Not-worrying and Not-distracting were excluded, making a comparison with our results impossible. In the Turkish version, a six-factor solution was only reached after removing items 1, 6, 19, 20, and 35. We refrained from excluding items to be able to make adequate comparisons between the MAIA-2-NL and the original MAIA-2. In our opinion adjustments to existing questionnaires should be made with caution and parsimony because they negatively influence the comparison between studies.

A possible explanation for the aggregation of the two original subscales Noticing and Emotional awareness in our factor analysis could be that the subscales both measure awareness, although differing in the cognitive appraisal being measured. Several items of the subscale Noticing and Emotional awareness show some overlap in sentence formulation, for example, item 1, subscale Noticing (“When I am tense, I notice where the tension is located in my body”) and item 23, subscale Emotional Awareness (“I notice how my body changes when I am angry”).

The original MAIA-2 subscales Self-regulation and Body listening are also considered one factor in our analysis. This could be explained by the fact that both subscales are described by Mehling et al. [17] as belonging to an overarching dimension of Mind-Body Integration, referring to an overall felt sense of an embodied self. This implies that one has access to more developed levels of body awareness, to a sense of “the interconnectedness of mental, emotional, and physical processes as opposed to a disembodied sense of alienation and of being disconnected from one’s body” [17, p.3].

Although we retained six factors in the EFA for the MAIA-2-NL versus eight factors in the original MAIA-2 [17], it should be noted that the two ‘new’ factors consist of four ‘old’ factors taken together two by two. More important, the CFA based on the original eight factors shows a better fit than the CFA based on six factors. These considerations lead to the conclusion that, with the present knowledge, maintaining eight factors isacceptable. Especially when comparing study results on the MAIA-2-NL with other studies, using eight factors, as in the original English scale, seems preferable. It would ofcourse be advisable to replicate the EFA and CFA in another large population sample.

This study has a couple of unique strengths. First, it benefits from a large sample size, setting it apart from other studies in the field. This large sample size offered the possibility of randomly splitting the sample in half and conducting EFA on one half of the sample and CFA on the other, as recommended by Swami and Barron [39]. It should be noted that, unfortunately, a considerable amount of the studies on the psychometric properties of the MAIA-2 were conducted in relatively small samples. For example, the study on the Turkish version of the MAIA-2 [21] was conducted in a total sample of 400 respondents, which is on the small side when taking into account the recommended participant-to-item ratio of 10:1 for EFA [36, 39]. The same goes for the French validation of the MAIA-2 [20], based on an EFA in 154 respondents and a CFA in another 154 respondents.

Another strong point lies in the insight provided by this study into the temporal reliability of the MAIA-2-NL, which ranges from moderate to good. As test-retest reliability is one of the components of the Reliable Change Index, this is essential knowledge in view of future studies on the evaluation of therapeutic interventions addressing interoceptive awareness.

Lastly, due to the digital administration of the questionnaire, no missing data were present. Additionally, this method eliminates the possibility of input errors that could occur with pen and paper administration, which could have implications for data accuracy.

The study has some limitations that need to be addressed in future research. First, construct validity for the MAIA-2-NL has not yet been investigated. Furthermore, as mentioned by Todd et al. [45], men and older adults often remain underrepresented in this kind of studies. This is, regrettably, also the case in our study. The mean age in our convenience sample was 35 years and men comprised one-third of the sample. It is important to note that the gender distribution is not equal and thus does not reflect the gender composition of the general Dutch population. Nevertheless, compared with other studies with much smaller male datasets [e.g., 20, 21, 23, 24], this study includes a substantial number of male participants (*n* = 331), thus contributing valuable comparative data. It is evident that more data from an elderly group and male respondents need to become available in order to speak not of a convenience sample but of a sample representing the Dutch population.

More importantly, future studies need to be conducted in clinical samples. This is, of course, an essential step in order to be able to evaluate problems in interoceptive awareness in different groups of patients and in order to measure the effect of interventions targeting these problems. However, confirmatory factor analyses in these samples should be performed before drawing conclusions on scores obtained with the MAIA-2 in clinical samples [19]. Such a strict psychometric procedure is worthwhile and should preferably be followed because it provides a sound base for further usage of an instrument. Unfortunately, obtaining the required sample size in a clinical setting is often challenging. An exception is the German version of the MAIA-2, validated in a sample of severely depressed patients (*n* = 110). Importantly, The MAIA-2 was found reliable and sensitive to change in this sample [5].

To summarize, our findings indicate that the MAIA-2-NL is a reliable and valid instrument to measure interoceptive awareness in a non-clinical sample of Dutch adults.

### Electronic supplementary material

Below is the link to the electronic supplementary material.

Supplementary Material 1

## Data Availability

The dataset used in the current study is available from the corresponding author upon reasonable request.
