# Supplementary material for: The Multidimensional Assessment of Interoceptive Awareness, version 2 (MAIA-2): psychometric properties in a Dutch non-clinical sample
Source: BMC Psychol. 2024 Jan 29;12:53. doi: 10.1186/s40359-024-01553-8 (PMC10826081; doi:10.1186/s40359-024-01553-8)
Supplement: Supplementary file 1 — Supplementary Material 1 [file 40359_2024_1553_MOESM1_ESM.pdf]

Dutch version Supporting Information

S1 Table 1. Multidimensional Assessment of Interoceptive Awareness 2, Dutch version

1.a	Als ik gespannen ben, voel ik waar in mijn lichaam de spanning zit
2.a	Ik merk het als ik niet lekker in mijn vel zit
3.a	Ik merk waar ik me in mijn lichaam lekker voel
4.a	Ik merk het als mijn ademhaling verandert, bijvoorbeeld of ik langzamer of sneller ga ademen
5.b	Lichamelijke spanning of ongemak merk ik niet op (negeer ik) tot het veel erger wordt (R)
6.b	Ik leid mezelf af van gevoelens van ongemak (R)
7.b	Bij pijn of ongemakken tracht ik op mijn tanden te bijten en door te zetten (R)
8.b	Ik probeer pijn te negeren (R)
9.b	Ik zet gevoelens van ongemak van me af door me op iets anders te richten (R)
10.b	Als ik onplezierige lichamelijke sensaties heb, ga ik me met iets anders bezig houden, zodat ik het niet hoef te voelen (R)
11.c	Wanneer ik lichamelijke pijn voel, raak ik van streek (R)
12.c	Als ik maar een beetje last van iets heb, ga ik me zorgen maken dat er iets mis is (R)
13.c	Ik kan een onaangenaam gevoel in mijn lijf opmerken zonder me er zorgen over te maken
14.c	Bij ongemak of pijn kan ik rustig blijven en me geen zorgen maken
15.c	Als ik ongemak of pijn ervaar, dan blijf ik er mee bezig (R)
16.d	Ik kan op mijn ademhaling letten zonder te worden afgeleid door wat er om me heen gebeurt
17.d	Ik kan me bewust blijven van wat ik in mijn lichaam voel, zelfs als er om me heen van alles gebeurt
18.d	Als ik met iemand in gesprek ben, kan ik aandacht schenken aan mijn houding
19.d	Als ik afgeleid ben, kan ik mijn aandacht weer terugbrengen naar mijn lichaam
20.d	Ik kan mijn aandacht verschuiven van denken naar het voelen van mijn lijf
21.d	Ik kan me bewust blijven van mijn hele lichaam, zelfs als ik ergens pijn heb of ongemak voel
22.d	Ik kan me bewust richten op mijn lichaam als geheel
23.e	Ik merk hoe mijn lichaam verandert als ik boos ben
24.e	Als er iets mis is in mijn leven, kan ik dat aan mijn lichaam voelen
25.e	Ik merk dat mijn lichaam anders voelt na een rustgevende ervaring
26.e	Ik merk dat mijn ademhaling vrij en gemakkelijk wordt als ik me op mijn gemak voel

27.e	Ik merk hoe mijn lichaam verandert wanneer ik me gelukkig / vrolijk voel
28.f	Als er te veel op me afkomt, kan ik een rustige plek in mezelf vinden
29.f	Als ik de aandacht op mijn lichaam richt, krijg ik een gevoel van rust
30.f	Ik kan mijn ademhaling gebruiken om spanning te verminderen
31.f	Als ik gevangen zit in gedachten, kan ik mijn geest tot rust brengen door me op mijn lichaam/ademhaling te concentreren
32.g	Ik luister naar informatie die mijn lichaam me over mijn emotionele toestand geeft
33.g	Wanneer ik van streek ben, neem ik de tijd om na te gaan hoe mijn lichaam aanvoelt
34.g	Ik luister naar mijn lichaam om te weten wat ik moet doen
35.h	Ik voel me thuis in mijn lichaam
36.h	Mijn lichaam voelt als een veilige plek
37.h	Ik vertrouw op wat ik in mijn lijf voel

R = reversely scored; a = subscale Noticing; b = subscale Not-distracting; c = subscale Not-worrying; d = subscale Attention regulation, e= subscale Emotional awareness, f = subscale Self-regulation, g = subscale Body listening; h = subscale Trusting.

Multidimensional Assessment of Interoceptive Awareness (MAIA)
Version 2
(2018)

Contact: Wolf E. Mehling, MD
Osher Center for Integrative Medicine
University of California, San Francisco
1545 Divisadero St., 4th floor
San Francisco, CA 94115
Phone: 01 (415) 353 9506
Wolf.Mehling@ucsf.edu
https://osher.ucsf.edu/maia/

Multidimensional Assessment of Interoceptive Awareness

Permission and Copyright

Although the MAIA survey is copyrighted, it is available without charge and no written permission is required for its use. This assumes agreement with the following as a consequence of using a MAIA survey:

- Please refer to the survey using its complete name – Multidimensional Assessment of Interoceptive Awareness - and provide the appropriate citation.
- Modifications may be made without our written permission. However, please clearly identify any modifications in any publications as having been made by the users. If you modify the survey, please let us know for our records.
- We recommend including entire subscales when selecting items from the MAIA to retain the psychometric features of these subscales (rather than selecting items from subscales).
- If you translate the MAIA into another language, please send us a copy for our records.
- If other investigators are interested in obtaining the survey, please refer them to the source document (PLoS-ONE 2012, and www.osher.ucsf.edu/maia/) to assure they obtain the most recent version and scoring instructions.

Scoring Instructions

Take the average of the items on each scale.
Note: (R): reverse-score (5 – x) items 5, 6, 7, 8, 9 and 10 on Not-Distracting, and items 11, 12 and 15 on Not-Worrying.

1. **Noticing:** Awareness of uncomfortable, comfortable, and neutral body sensations

   Q1______ + Q2______ + Q3______ + Q4______ / 4 = ___________

2. **Not-Distracting:** Tendency not to ignore or distract oneself from sensations of pain or discomfort

   Q5(**R**)____ + Q6(**R**)____+ Q7(**R**)____+ Q8(**R**)____+Q9(**R**)____+Q10(**R**) / 6 = ___________

3. **Not-Worrying:** Tendency not to worry or experience emotional distress with sensations of pain or discomfort

   Q11(**R**)______ + Q12(**R**)______ + Q13______ + Q14______ + Q15 (**R**) / 5 = ___________

4. **Attention Regulation:** Ability to sustain and control attention to body sensations

   Q16_____ + Q17_____ + Q18_____ + Q19_____ + Q20_____ + Q21_____ + Q22_____ / 7 = ________

5. **Emotional Awareness:** Awareness of the connection between body sensations and emotional states

   Q23_____ + Q24_____ + Q25_____ + Q26_____ + Q27_____ / 5 = ___________

6. **Self-Regulation:** Ability to regulate distress by attention to body sensations

   Q28_____ + Q29_____ + Q30_____ + Q31_____ / 4= ___________

7. **Body Listening:** Active listening to the body for insight

   Q32_____ + Q33_____ + Q34_____ / 3= ___________

8. **Trusting:** Experience of one's body as safe and trustworthy

   Q35_____ + Q36_____ + Q37_____ / 3= ___________

Below you will find a list of statements. Please indicate how often each statement applies to you generally in daily life.

.	Circle one number on each line					
	**Never**					**Always**
1. When I am tense I notice where the tension is located in my body.	0	1	2	3	4	5
2. I notice when I am uncomfortable in my body.	0	1	2	3	4	5
3. I notice where in my body I am comfortable.	0	1	2	3	4	5
4. I notice changes in my breathing, such as whether it slows down or speeds up.	0	1	2	3	4	5
5. I ignore physical tension or discomfort until they become more severe.	0	1	2	3	4	5
6. I distract myself from sensations of discomfort.	0	1	2	3	4	5
7. When I feel pain or discomfort, I try to power through it.	0	1	2	3	4	5
8. I try to ignore pain	0	1	2	3	4	5
9. I push feelings of discomfort away by focusing on something	0	1	2	3	4	5
10. When I feel unpleasant body sensations, I occupy myself with something else so I don't have to feel them.	0	1	2	3	4	5
11. When I feel physical pain, I become upset.	0	1	2	3	4	5
12. I start to worry that something is wrong if I feel any discomfort.	0	1	2	3	4	5
13. I can notice an unpleasant body sensation without worrying about it.	0	1	2	3	4	5
14. I can stay calm and not worry when I have feelings of discomfort or pain.	0	1	2	3	4	5
15. When I am in discomfort or pain I can't get it out of my mind	0	1	2	3	4	5
16. I can pay attention to my breath without being distracted by things happening around me.	0	1	2	3	4	5
17. I can maintain awareness of my inner bodily sensations even when there is a lot going on around me.	0	1	2	3	4	5
18. When I am in conversation with someone, I can pay attention to my posture.	0	1	2	3	4	5

How often does each statement apply to you generally in daily life? Circle one number on each line

	Never					Always
19. I can return awareness to my body if I am distracted.	0	1	2	3	4	5
20. I can refocus my attention from thinking to sensing my body.	0	1	2	3	4	5
21. I can maintain awareness of my whole body even when a part of me is in pain or discomfort.	0	1	2	3	4	5
22. I am able to consciously focus on my body as a whole.	0	1	2	3	4	5
23. I notice how my body changes when I am angry.	0	1	2	3	4	5
24. When something is wrong in my life I can feel it in my body.	0	1	2	3	4	5
25. I notice that my body feels different after a peaceful experience.	0	1	2	3	4	5
26. I notice that my breathing becomes free and easy when I feel comfortable.	0	1	2	3	4	5
27. I notice how my body changes when I feel happy / joyful.	0	1	2	3	4	5
28. When I feel overwhelmed I can find a calm place inside.	0	1	2	3	4	5
29. When I bring awareness to my body I feel a sense of calm.	0	1	2	3	4	5
30. I can use my breath to reduce tension.	0	1	2	3	4	5
31. When I am caught up in thoughts, I can calm my mind by focusing on my body/breathing.	0	1	2	3	4	5
32. I listen for information from my body about my emotional state.	0	1	2	3	4	5
33. When I am upset, I take time to explore how my body feels.	0	1	2	3	4	5
34. I listen to my body to inform me about what to do.	0	1	2	3	4	5
35. I am at home in my body.	0	1	2	3	4	5
36. I feel my body is a safe place.	0	1	2	3	4	5
37. I trust my body sensations.	0	1	2	3	4	5

MAIA ©2018, University of California San Francisco https://osher.ucsf.edu/maia/
